# Acute endocarditis complicated by left ventricular pseudoaneurysm with acute bloody pericardial effusion: A case report

**DOI:** 10.1097/MD.0000000000036233

**Published:** 2023-12-08

**Authors:** Akie Shimada, Taira Yamamoto, Shizuyuki Dohi, Yasutaka Yokoyama, Daisuke Endo, Minoru Tabata

**Affiliations:** a Department of Cardiovascular Surgery, Nerima Hospital, Juntendo University, Tokyo, Japan; b Department of Cardiovascular Surgery, Juntendo University, Tokyo, Japan.

**Keywords:** annular abscess, cerebral infarction, infective endocarditis, skin eruption, vegetation, ventricular pseudoaneurysm

## Abstract

**Rationale:**

Delay in seeking medical attention for high fever and inadequate diagnosis can lead to rapid progression of inflammation and spread to surrounding tissues and organs. *Staphylococcus aureus* is a common cause of systemic infections, and infectious endocarditis can swiftly become severe; therefore, careful management is required.

**Patient concerns:**

A 54-year-old woman was admitted to our hospital with high fever and progressive loss of consciousness. Meningitis was suspected, and antibiotic treatment was initiated. Blood culture revealed methicillin-sensitive *Staphylococcus aureus.* Subsequently, the patient developed hypotension, bradycardia, and cardiac arrest and underwent emergency cardiopulmonary resuscitation.

**Diagnoses:**

Transesophageal echocardiography performed during the procedure revealed significant vegetation at the posterior leaflet of the mitral valve, an abscess at the valve annulus, and a pseudoaneurysm of the left ventricular posterior wall.

**Interventions:**

The patient underwent emergency small incision pericardiotomy drainage, and her blood pressure and heart rate stabilized. After pericardial drainage, acute renal failure, fulminant hepatitis, and disruption of coagulation function were observed, and she was treated with plasma exchange therapy and intravenous immunoglobulin. Resection of the huge vegetation, debridement, patch closure of the ventricular perforation, and mitral valve replacement were performed.

**Outcomes:**

Surgical findings showed massive vegetation in the posterior leaflet of the mitral valve, an annular abscess in the posterior leaflet of the mitral valve connected to the left ventricular posterior wall, and a pseudoaneurysm. Postoperatively, her pseudoaneurysm resolved and her cardiac function stabilized, while circulatory failure due to bacteremia progressed, and she gradually developed acidosis and unstable blood pressure. Plasma exchange and continuous hemodiafiltration were continued; however, she died of progressive multiorgan failure.

**Lesson:**

*Staphylococcus aureus* bacteremia can cause fatal complications. Even when symptoms of meningitis are suspected, it is essential to examine the patient for endocarditis. Delayed diagnosis can lead to fatal endocarditis-related complications.

## 1. Introduction

Even with many advances in diagnostic techniques, pitfalls still exist in diagnosing fever of unknown origin, and delays in a proper diagnosis and treatment can lead to severe complications. In developed countries, *Staphylococcus aureus (S. aureus*) is an important and growing cause of infective endocarditis (i.e.),^[1,2]^ which can rapidly progress and spread to surrounding tissues and organs.^[3]^ *S. aureus* causes most abscesses in pericarditis; however, it tends to be more severe in immunocompromised patients with atopic dermatitis or after cancer treatment.

Although valvular abscesses can occur as a complication of i.e., reports of the breakdown and perforation of left ventricular structures to form a pseudoaneurysm are extremely rare.^[4]^ Pseudoaneurysms have a high risk of rupture, forming a fistula in the left atrium that can decompress or cause hemorrhagic cardiac tamponade if it ruptures. This condition causes a sudden hemodynamic collapse and is difficult to treat without emergency surgical intervention. Especially in a state of septic shock, when excessive vasodilation occurs and blood pressure is low, rupture of a pseudoaneurysm and rapid cardiac tamponade can result in a catastrophic condition with no output through the heart as blood circulation. Depending on the location and size of the pseudoaneurysm, it has also been reported that it can compress coronary arteries and cause myocardial infarction.^[5,6]^ Left ventricle (LV) pseudoaneurysms are relatively easy to detect with echocardiography and can lead to early surgical treatment if adequately diagnosed by a cardiologist at the appropriate time. However, physicians sometimes encounter patients who do not seek medical attention for a persistent high fever and finally visit the emergency room only after the onset of impaired consciousness or multiorgan complications. In such cases, many physicians may suspect other diseases, which may delay a cardiologist’s evaluation. We report a tragic case of i.e. in which a *S. aureus* abscess rapidly progressed to an LV pseudoaneurysm.

## 2. Case presentation

A 54-year-old woman had been prescribed anti-inflammatory and analgesic medications at a local clinic because of back pain and general fatigue for 1 week. However, her high fever persisted, and she was taken by a family member to the emergency room when she developed a loss of consciousness with no memory for approximately 2 days. Her medical history included surgery and radiation therapy for left breast cancer 10 years before. She had no history of cardiac disease, recent dental treatment, drug abuse, or continuous medical treatment. The patient also presented with atopic dermatitis.

On admission, the patient was febrile (38.0°C), tachycardic (102 beats/min [bpm]), and hypotensive (86/52 mm Hg). She was highly disoriented and confused. The patient also had mild cervical stiffness but no paralysis of the extremities. Her neck flexion test was negative, Kernig’s sign was negative, and her neurological examination was normal. The patient’s breath sounds were clear, and no heart murmur was heard. Chronic eczema due to atopic dermatitis was present on the medial elbows of both upper extremities; however, there were no other skin manifestations and no findings strongly suggestive of pneumonia or infective endocarditis.

**Figure 1.**
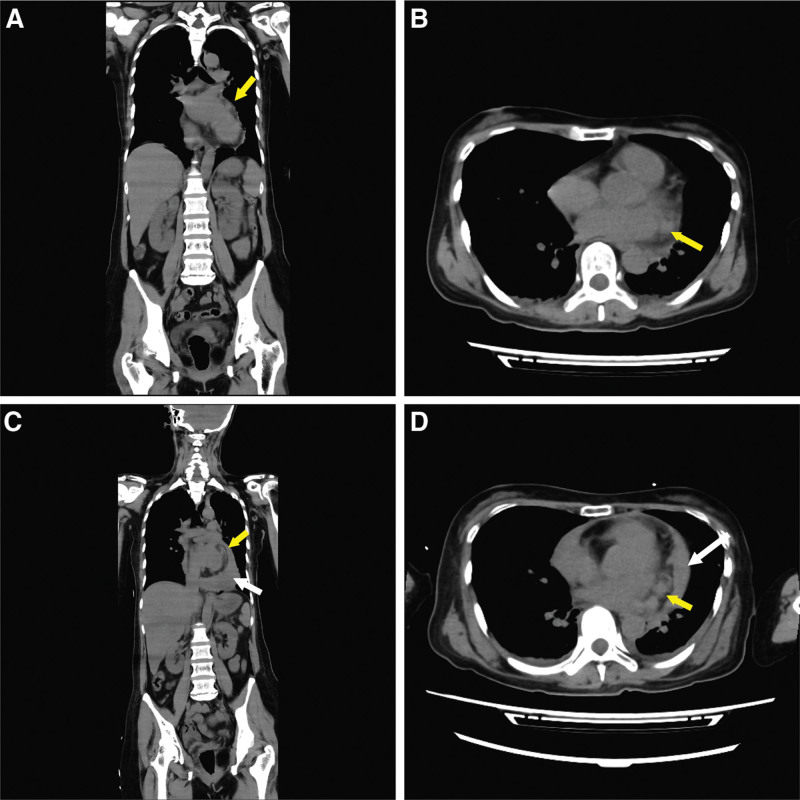
A. Preoperative computed tomography (CT) scan taken at admission. The preoperative sagittal CT image shows a mass in the pericardial space at the posterior wall of the left ventricle (yellow arrow). B. Preoperative CT scan taken at admission. The preoperative horizontal CT image shows a mass in the pericardial space at the lateral wall of the left atrium and below the left atrial appendage (yellow arrow). C. Preoperative CT scan taken after cardiopulmonary resuscitation. The preoperative sagittal CT image after cardiopulmonary resuscitation shows a mass in the pericardial space on the posterior wall of the left ventricle (yellow arrow) and pleural effusion (white arrow). D. Preoperative CT scan taken after cardiopulmonary resuscitation. The preoperative horizontal CT image after cardiopulmonary resuscitation shows a mass in the pericardial space at the posterior wall of the left ventricle (yellow arrow) and pleural effusion (white arrow).

Preoperative laboratory results are shown in Table 1. The patient’s brain computed tomography (CT) scan showed normal findings with no hemorrhage or infarction. Chest radiography revealed an enlarged heart (cardiothoracic ratio of 61.8%) and pulmonary congestion. Thoracoabdominal CT scans showed no enlarged organs or lymph nodes and no abscess, bleeding, or infarction areas. In retrospect, a small mass was observed posterior to the LV (Fig. 1A and B). However, this small mass was not considered an abnormal finding at the time of emergency admission because meningitis was strongly suspected. The electrocardiogram showed a normal sinus rhythm (100 bpm) and no ST-T changes. The patient’s white blood cell count and C-reactive protein count were markedly increased, and her platelet count was decreased to 2.1 × 10^9^/L. Hepatic and renal dysfunction were mild; however, albumin was significantly reduced and severe infection, especially bacteremia, was strongly suspected. Meningitis with disseminated intravascular coagulation syndrome (DIC) was suspected because of impaired consciousness, and the neurological team initiated medical treatment. They avoided lumbar puncture for close examination of meningitis because of the low platelet count.

**Table 1:** Changes in tests after hospitalization.

	Day 0 (at admission)	Day 0 (after CPR)	Day 1	Day 3	Day 7
Blood pressure (mm Hg)	87/52	88/66	122/80	122/90	110/72
Heart rate (bpm)	104	100	90	70	66
Body temperature	37.9	37.8	36.0	36.7	37.2
White blood cells (×10^3^/L)	18.4	39.0	22.9	19.1	31.8
PT-INR	1.18	1.58	1.54	1.70	1.29
aPTT (s)	29.9	35.5	33.0	29.2	37.5
AST (U/L)	88	302	1717	3686	194
ALT (U/L)	94	107	638	1827	446
CK (U/L)	371	1924	17908	2134	127
Total bilirubin (mg/dL)	1.90	1.30	2.80	5.50	4.70
Direct bilirubin (mg/dL)	1.00	1.00	1.90	3.90	3.30
Total protein (g/dL)	5.7	4.6	5.0	7.5	7.6
Albumin (g/dL)	2.3	1.8	3.3	3.9	3.6
C-reactive protein (mg/dL)	36.51	33.54	26.18	11.70	5.81
Lactate (mmol/L)	3.3	24.0	22.0	6.5	2.1
Complement component 3 (mg/dL)	46				
Complement component 4 (mg/dL)	13				
PR3-ANCA (U/mL)	<5.0				
MMP-3 (ng/mL)	60.9				

ALT = alanine aminotransferase, aPTT = activated partial thromboplastin time, AST = aspartate aminotransferase, CK = creatine kinase, CPR = cardiopulmonary resuscitation, MMP-3 = Matrix metalloproteinase 3, PR3-ANCA = proteinase-3- antineutrophil cytoplasmic antibodies, PT-INR = prothrombin time-international normalized ratio.

After admission, the patient’s hypotension (88/60 mm Hg) and tachycardia (100 bpm) remained unchanged, and she was diagnosed with septic shock in a state of metabolic acidosis (pH, 7.150; paCO_2_, 29.3 mm Hg; base excess, −17.3 mmol/L; lactic acid, 24 mmol/L). Continuous intravenous infusion of noradrenaline was initiated.

Blood cultures revealed methicillin-sensitive *S. aureus* at 12 hours. Vancomycin and ceftriaxone sodium hydrate were administered. Fourteen hours after admission, the patient’s heart rate dropped from 100 to 40 bpm, and her blood pressure was not palpable; therefore, emergency cardiopulmonary resuscitation was performed. After resuscitation, the patient’s blood pressure recovered to 90 mm Hg, and a CT scan revealed pericardial effusion (Fig. 1C and D). Transthoracic echocardiography (TTE) findings were suspect for bloody pericardial effusion, infective endocarditis of the mitral valve, and mitral annular abscess. Because the patient was in DIC, pericardial drainage was performed through a small subxiphoid incision instead of a puncture. The pericardial effusion was 500 mL and bloody. Pericardial fluid analysis revealed 27,600 white blood cells (98% polymorphonuclear cells) and a hemoglobin level of 9.4 g/dL. Cytological, histological, and mycobacterial cultures revealed no malignant pericardial effusion or tuberculous pericarditis. After emergency admission, TTE was delayed because a heart murmur was not heard.

**Figure 2.**
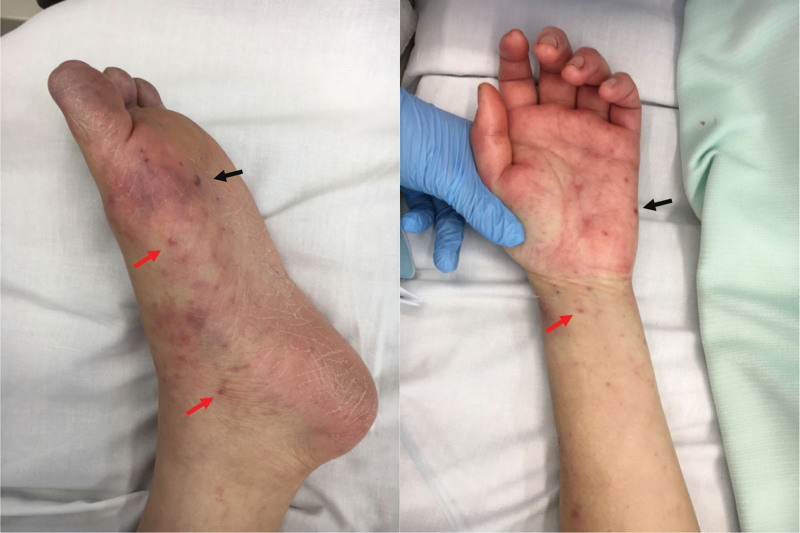
Osler nodules (black arrow) and Janeway lesions (red arrow) are on the lower and upper limbs.

**Figure 3.**
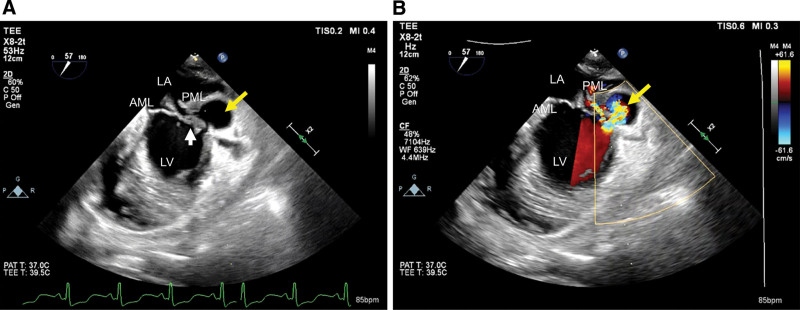
A. Transesophageal cardiac ultrasonography shows the pseudoaneurysm (yellow arrow). The vegetation on the posterior mitral leaflet (white arrow) is shown. B. Transesophageal cardiac ultrasonography image shows the pseudoaneurysm (yellow arrow). The vegetation on the posterior mitral leaflet (white arrow) and the shunt (yellow arrow) are indicated. AML = anterior mitral leaflet, LA = left atrium, LV = left ventricle, PML = posterior mitral leaflet.

The patient’s blood pressure recovered after removing the tamponade; however, her liver function and DIC status worsened, and we determined that many organs, including the liver and kidneys, would not tolerate emergency open heart surgery. In addition, after cardiopulmonary resuscitation, the patient was highly disoriented, and her response to pain disappeared. Therefore, plasma exchange and intravenous immunoglobulin therapy were performed to treat multiple organ failure and DIC. Serum immunological antibody tests were normal. Acute renal failure also improved postoperatively, and her liver and coagulation functions recovered after 1 week. However, skin examination revealed a new rash daily (Fig. 2). TTE findings showed good LV function. Transesophageal echocardiography (TEE) was not performed because of the severe disturbance of consciousness and bleeding tendency due to fulminant hepatitis and DIC, with the focus being on the treatment of the liver and kidneys. However, we performed TEE on day 6 because the mitral valve vegetation and the mitral annular abscess were enlarged. TEE revealed extensive vegetation at the posterior leaflet of the mitral valve, a pseudoaneurysm on the posterior wall of the LV, and an abscess at the annulus (Fig. 3A and B). The patient’s i.e. deteriorated progressively. Although the patient had prolonged impaired consciousness, emergency surgery was performed because her head CT scans showed no signs of cerebral infarction, cerebral hemorrhage, or hypoxic encephalopathy.

**Figure 4.**
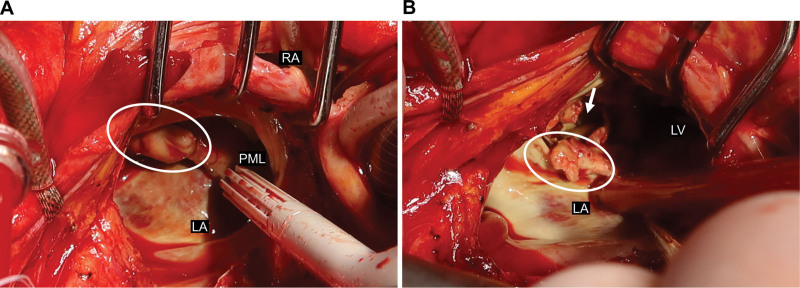
A. Operative findings. Intraoperative view shows the vegetation (white oval). B. Operative findings. The intraoperative view shows vegetation on the mitral annulus (white oval) and pseudoaneurysm (white arrow) after the resection of the PML and vegetation. LA = left atrium, LV = left ventricle, PML = posterior mitral leaflet, RA = aright atrium.

Emergency surgery was performed through a standard median sternotomy. Artificial cardiopulmonary bypass was established with the ascending aorta as the inflow channel, and the superior and inferior vena cava as the outflow channels. The vent tube into the left atrium was inserted after aortic clamping because of the presence of vegetation. Surgical findings showed that the abscess almost wholly covered the posterior leaflet of the mitral valve, and P1 and P2 had disappeared due to tissue destruction. Because of the massive vegetation, there was no significant regurgitation due to mitral valve insufficiency. The valve annulus abscess cavity perforated the posterior LV wall and formed a pseudoaneurysm (Fig. 4A and B). A culture of pus from the abscess cavity revealed methicillin-sensitive *S. aureus*. We closed the perforation with bovine pericardium (Edwards Life Sciences, Irvine, CA) and the prosthetic graft (J-graft; Japan Lifeline Co., Ltd., Tokyo, Japan) using nodal and continuous sutures for double-reinforcement closure. We then augmented the valve annulus at the posterior leaflet of the mitral valve. The mitral valve was replaced with a prosthetic mechanical valve (On-X mitral heart valve; Artivion, Inc., Kennesaw, GA). Postoperative recovery of cardiac function was good, and the pseudoaneurysm disappeared. The operation time was 240 minutes, the cardiopulmonary bypass time was 183 minutes, and the aortic clamping time was 150 minutes. However, the patient was refractory to antibiotic therapy. Postoperative continuous hemodiafiltration and concomitant treatment were used; however, liver and renal failure progressed again in 1 week, metabolic acidosis became pronounced, the hemodynamic status could not be maintained, and the patient could not be saved.

## 3. Discussion

In developed countries, i.e. caused by *S. aureus* is increasing and tends to be particularly severe in immunocompromised cases of atopic dermatitis and in immunocompromised patients such as after cancer treatment. Delayed detection of i.e. caused by *S. aureus* can lead to valve annular abscesses and pseudoaneurysms, which require early detection and treatment to prevent mortality.^[7]^ When a simple *S. aureus* infection becomes antibiotic-resistant or treatment is delayed, the perivalvular abscess is destroyed over time (due to fibrous tissue necrosis) and it loses its typical structure.^[8]^ This destroyed and perforated area may communicate with high-pressure chambers such as the LV and aorta, and pseudoaneurysms leading to high-pressure system tissue are prone to saccular dilation and can perforate.^[9]^ Hemodynamic instability due to septic shock and low cardiac output due to cardiac tamponade can lead to fatal circulatory failure.

LV pseudoaneurysms due to mitral valve i.e. account for < 1% of all cases; however, the risk of rupture is 35% to 40%, and rupture can be fatal.^[10]^ LV pseudoaneurysms usually arise from a mitral valve-aortic mesenteric abscess and project externally between the aortic root and the left atrium. In contrast, LV posterior wall lesions are rare.^[11]^

Owing to the delayed diagnosis in this patient, a huge abscess covered the mitral valve, preventing the auscultation of a mitral regurgitation murmur. Owing to the delayed treatment, the pseudoaneurysm spread to the outer LV. The pseudoaneurysm most likely ruptured during the intense cardiopulmonary resuscitation performed when the patient experienced cardiac arrest due to hypotension.

Imaging studies have been reported as useful in cases of i.e. with abscesses and delayed pseudoaneurysms. Cardiac CT and magnetic resonance imaging can effectively diagnose posterior cardiac wall pseudoaneurysms. Although TEE highlights abscess formation and pseudoaneurysm formation in the posterior wall of the LV from the valve annulus, electrocardiogram-synchronized cardiac three-dimensional computed tomography can also be used to identify surrounding structures such as the coronary circumflex artery and coronary sinuses. Cardiac magnetic resonance imaging is the best preoperative imaging test for characterizing aneurysm wall organization and can distinguish between pseudoaneurysms and true aneurysms. However, these tests can only be performed under stable conditions. Performing them under precarious hemodynamic or coagulation disruptive conditions may lead to new complications or sudden deterioration during the examination. In previous reports, imaging diagnosis was not completed in the acute phase; however, an accurate, definitive diagnosis was obtained by performing the tests mentioned previously under stable hemodynamic conditions. In addition, in these reports, the surgical team was informed of the need for surgery immediately after imaging was obtained, and the emergency surgery was performed with promising results.^[12–14]^

In this patient, a pseudoaneurysm should have been suspected from the outset because plain CT scan on admission revealed an abnormal mass in the posterior LV wall. Pseudoaneurysms can form a fistula in the left atrium, decompress or perforate the epicardium, and bleed into the pericardial sac, thereby causing cardiac tamponade and immediate death. The size, location, and extent of the structural abnormality may cause coronary artery compression, resulting in acute myocardial infarction.^[15]^ These complications are infrequent; however, they are associated with high mortality rates if not detected and treated promptly.^[4]^ In the current coronavirus disease 2019 era, fever of unknown origin tends to be diagnosed late. For persistent and treatment-resistant fever, timely echocardiography can prevent severe complications.

Infective endocarditis can cause a variety of histological complications. Fatal cardiac structural complications occur when the infection spreads into the heart and produces a valvular abscess or pseudoaneurysm.^[16]^ Pseudoaneurysm formation is thought to arise from an extravalvular abscess during remodeling, forming always in communication with a high-pressure chamber such as the left chamber or the aorta. It has been thought to occur when a weakened abscess undergoes necrosis because of its constant connection to high-pressure chambers such as the LV and aorta.^[4]^ In the LV, pseudoaneurysms are reported to arise from mitral-aortic intervalvular fibrosa abscesses, which usually occur in the anterior wall.^[17]^ However, they can also form on the posterior wall such as in the present case in which highly invasive staphylococcus bacteria caused a vegetation and abscess on the posterior leaflet of the mitral valve. However, this situation is less frequently reported.^[18]^ The inflammatory material associated with the infection is thought to induce myocardial tissue necrosis, weakening the myocardial wall structure and causing dissection of the structure.^[6]^ The theory of extravalvular abscess reformation has been reported because the opening of a pseudoaneurysm resembles a slit or dissection into the myocardium.^[6]^ It has been reported that the significant contents of pseudoaneurysms are mainly thrombus with virtually no bacterial masses or inflammatory cells and negligible neutrophil infiltration.^[6]^ Our patient had nearly the same findings.

*S. aureus* infection was an independent predictor of 1-year mortality in patients with left-sided native valve infective endocarditis and intracardiac abscess and left ventricular ejection fraction < 40% were independent risk factors for in-hospital mortality during surgical treatment. In addition, intracardiac abscess and valve perforation were reported as independent risk factors for 1-year posttreatment mortality.^[19]^ Abscesses are life-threatening complications that cannot be cured by antibiotic therapy alone. Intracardiac abscesses, valve perforation, or extensive tissue destruction are indications for early surgery in i.e. The association between extensive cardiac infection and adverse outcomes may be related to uncontrolled infection or increased *S. aureus* virulence. The development of fatal fulminant hepatitis, disruption of coagulation function, or impaired consciousness with no recovery unfortunately make surgical timing more difficult. Future studies on microorganisms and host-specific factors are warranted to further elucidate the pathogenesis of *S.* bacteremia. Establishing independent echocardiographic markers and CT observations as emergency tests in sepsis should ideally lead to more aggressive patient management in patients with septic shock.

This report describes a sporadic case of ruptured LV pseudoaneurysm during septic shock in a patient receiving treatment for meningitis. In this case, a simple CT image obtained at admission showed evidence of a left ventricular pseudoaneurysm. Such findings can support future imaging and treatment decisions in the emergency setting for similar cases. The limitations of this case include the fact that the emergency department staff neither suspected an i.e. nor did they detect the mass on the first CT scan. Consequently, the course of the rupture was unknown, and we could not report the remote stage of the pseudoaneurysm several years after treatment of the perforated area.

*S. aureus* bacteremia can develop under conditions of reduced skin immunity such as atopic dermatitis or postoperative radiotherapy for breast cancer. A delayed diagnosis can cause catastrophic endocarditis-related complications. This case review is highly relevant as it presents the imaging findings of the disease and raises awareness regarding its subtle clinical manifestations.

## Acknowledgments

We would like to thank Editage (www.editage.com) for English language editing.

## Author contributions

**Conceptualization:** Taira Yamamoto.

**Data curation:** Akie Shimada.

**Formal analysis:** Akie Shimada.

**Investigation:** Taira Yamamoto.

**Methodology:** Shizuyuki Dohi, Yasutaka Yokoyama, Daisuke Endo.

**Project administration:** Taira Yamamoto.

**Resources:** Taira Yamamoto.

**Supervision:** Minoru Tabata.

**Visualization:** Shizuyuki Dohi.

**Writing – original draft:** Akie Shimada.

**Writing – review & editing:** Taira Yamamoto.
